# Journeys to HIV testing and diagnosis among adults aged 50+ years in England: A qualitative interview study

**DOI:** 10.1177/1355819620943242

**Published:** 2020-12-02

**Authors:** Sadie Bell, Tim Doran, Fabiola Martin, Joy Adamson

**Affiliations:** 1 Research Fellow in Public Health Evaluation, Department of Global Health and Development, London School of Hygiene & Tropical Medicine, London, UK; 2 Professor of Health Policy, Department of Health Sciences, University of York, York, UK; 3 Consultant Physician in Sexual Heath Physician, HIV and HTLV-1 Medicine, Stonewall Medical Centre, Brisbane, Australia; 4 Senior Clinical Lecturer, School of Public Health, University of Queensland, Brisbane, Australia; 5 Mary Kinross Trust & Royal College of Surgeons Chair in Surgical Trials and Health Sciences, York Trials Unit, Department of Health Sciences, University of York, York, UK

**Keywords:** HIV, older people, HIV testing

## Abstract

**Objectives:**

In England, older adults (aged ≥50 years) are at greater risk of being diagnosed with advanced stage HIV infection than younger adults. We explored journeys to testing and diagnosis among older adults, examining factors associated with late HIV diagnosis in this age group.

**Methods:**

Semi-structured qualitative interviews were performed with 12 adults diagnosed with HIV at age 50+ years and 12 health care professionals working in sexual health/HIV services. Data were analysed thematically, using the Model of Pathways to Treatment as a framework for analysis.

**Results:**

Older adults were often found to experience non-linear and complex diagnostic journeys. Pathways to diagnosis were affected by 6 factors: (i) the non-specific nature of HIV symptoms and their misattribution as being age-related; (ii) symptom severity, impact, and visibility; (iii) HIV health literacy; (iv) perceptions of HIV risk; (v) geographical location; and (vi) assessment in non-specialist settings.

**Conclusions:**

Older adults appear to encounter additional barriers to HIV testing compared with younger people, particularly when they are not part of a group targeted in HIV prevention and testing campaigns. To diagnose HIV more promptly in adults aged 50+ years, HIV knowledge and risk perception must increase in both older people and health care professionals. Health care professionals need to look beyond the ‘high risk’ groups that are most affected by HIV and consider HIV more readily in the diagnostic process.

## Introduction

Across Europe, although rates of new HIV diagnosis are highest among younger people, new cases of HIV are rising in older adults (aged 50+ years) [1]. In the UK, between 2004 and 2015 new HIV diagnoses in the over 50 s increased from 3.1 to 4.3 cases per 100,000 people, during which time new cases in younger age groups declined [1]. In 2018, 21% of total new HIV diagnoses in the UK were reported in adults aged 50+ years (946 cases), compared to 6.7% in 2003 (487 cases) [2].

New HIV diagnoses in adults aged 50+ years are more likely to be made at a late stage of infection (CD4 T Lymphocyte count <350 cells/μl within 91 days of diagnosis) than in younger people [1,2]. 2017). Late HIV diagnosis is associated with increased morbidity and mortality risk [3], greater opportunity for onward HIV transmission [4], and higher health care costs [5]. Age is just one socio-demographic factor associated with late HIV diagnosis and rates also differ by sexual orientation, ethnicity, and geographical location. The probable exposure route of almost all new HIV infections in the UK, including those in older adults, is through sexual transmission [6]. The highest rates of late stage HIV diagnoses in the UK are reported in heterosexual males, Black African men and women, and people diagnosed outside of London [2].

To promote early HIV diagnosis, national guidelines for the UK recommend that testing should be offered across a range of primary, secondary, specialist sexual health, and community settings [7]. In areas of high HIV prevalence (≥2 cases per 1000 population aged 15–59 years) all general practice registrants and hospital admissions should be offered testing [7,8]. Despite these recommendations, an HIV test offer may be delayed due to a lack of health care professionals’ confidence and competency in test performance, or due to health care professionals’ perceptions of HIV risk [9]. Adults aged 40+ years have been found more likely to experience ‘missed presentations’. These are points at which individuals access health services, often on multiple occasions, with symptoms or clinical indicator conditions for HIV and health care professionals miss the opportunity to offer HIV testing [10]. In sexual health clinics, where testing should be universally offered, a decline in likelihood of test offer with advancing attendee age has been identified [11]. Patient factors linked to delays in seeking HIV testing include: fear of prognosis, HIV stigma and discrimination, lack of HIV knowledge and awareness, and low personal risk perception [9]. Older adults have been found more likely to present for testing at a late stage of disease [12,13].

To date, little UK-based research has specifically concentrated on adults diagnosed with HIV at age 50+ years and their health care-seeking journey [14]. This study explored journeys to testing among adults diagnosed with HIV at age 50+ years in England, examining barriers to prompt HIV diagnosis in this age group through interviews with service users and health care professionals working in HIV services.

## Methods

Semi-structured in-depth qualitative interviews were conducted with service users (SUs) diagnosed with HIV within the last 5 years at age 50+ years, and health care professionals (HCPs) working with older adults in NHS sexual health/HIV services.

### Study settings and recruitment

Service user (SU) recruitment took place at two NHS sites in areas categorized as having low HIV prevalence areas (<1 case per 1000 people) and two NHS sites in high HIV prevalence areas (>2 cases per 1000 people) at the time of data collection. The study was also advertised on the webpages of UK-based HIV charities. The recruitment approach aimed to maximize participant diversity in age (within the over 50 category), gender, ethnicity, and sexual orientation. SUs at NHS sites were invited to take part in the study by their sexual health/HIV clinician. Each approached individual was given an information sheet, detailing the study procedures and explaining all aspects of participation, including the right to withdraw from the research and issues around confidentiality and data protection. The study was advertised by UK-based HIV charities for SU recruitment using a poster.

HCP recruitment was targeted at two NHS sites used for SU recruitment, these being the lowest and highest HIV prevalence sites in the study. HCPs were identified through communication with clinicians already involved in SU recruitment and contacted via email by one of the authors (SB). Recruitment primarily focused on HCPs working in specialist sexual health/HIV nursing and medical roles.

### Participants

Twelve SUs and 12 HCPs were interviewed by SB. The first SU participant is referred to as “SU1”, the second as “SU2”, and so on (likewise with each HCP).

We aimed to achieve data saturation, where no new themes were emerging from the SU or HCP interviews; however, participant numbers were largely determined by how many volunteers were available during the time frame of the study. HCPs were recruited from nursing, medical, and psychology roles (Table 1). Most HCPs had worked in sexual health/HIV services for 10 or more years. HCP interviews took place either within sexual health/HIV settings or at the University of York.

**Table 1.** Health care professional participants.

Participant no.	Gender	Recruited from low or high HIV prevalence site	Job role
1	F	High	HIV specialist nurse
2	M	High	Associate specialist in HIV
3	M	High	HIV nurse
4	F	High	Clinical psychologist
5	F	Low	HIV lead nurse
6	F	High	HIV research murse
7	M	Low	Genito-urinary medicine (GUM) specialist nurse
8	M	Low	HIV consultant
9	M	Low	HIV lead nurse
10	F	Low	Speciality doctor in GUM
11	F	High	Nurse practitioner working in HIV
12	F	Low	Consultant in GUM

Most SUs were male (aside from SU2), identified themselves as men who have sex with men (MSM), and were diagnosed with HIV at 50–53 years (Table 2). SUs were mainly recruited from high HIV prevalence areas. Most SU interviews were conducted face-to-face, within HIV services or in participant's homes. One SU was recruited through a HIV charity and interviewed via telephone.

**Table 2.** Service user participants.

Participant no.	Gender	Age at diagnosis	Recruited from low or high HIV prevalence site	Approx. time after diagnosis that the interview took place	Sexual orientation	Test location	Missed opportunities for testing (presentation to health services before testing)	Initiator of HIV test	Late stage of HIV at diagnosis (yes/no)
1	M	67	High	7 months	Undisclosed	Hospital (inpatient)	No – tested for HIV as a direct hospital admission	HCP	Unclear – acuteness of symptoms indicative of a late diagnosis
2	F	55	High	11 months	Heterosexual	Hospital (A&E)	Attended GP 4 times and A&E 2 times. GP sent her to A&E with a note (2nd attendance). HIV test performed in A&E	HCP	No
3	M	53	High	1 year	Man who has sex with men (MSM)	Private GP	Attended GP and a hospital outpatient service several times before being tested at private GP practice	HCP	Yes
4	M	51	Low	1 year and 2 months	Heterosexual	Hospital (Intensive Care Unit - ICU)	Attended GP twice. Ambulance called at 2nd GP visit – admitted to hospital and tested in ICU	HCP	Yes
5	M	50	High	9 months	MSM	Sexual health clinic	No	Self-initiated	No
6	M	51	High	4 months	MSM	Sexual health clinic	No	Self-initiated	No
7	M	50	High	2 years	MSM	Sexual health clinic	No	Self-initiated	No
8	M	53	High	5 months	MSM	Hospital (ICU)	Attended GP on 3 occasions. On the 3rd GP visit an ambulance was called and he was admitted to hospital and tested in ICU.	HCP	Yes
9	M	53	High	4 months	MSM	Sexual health clinic	No	Self-initiated	No
10	M	57	High	2 years and 8 months	MSM	Hospital (inpatient)	Attended GP numerous times. Also referred to and seen by a Respiratory physician. Tested for HIV as an acutely unwell hospital admission	HCP	Yes
11	M	52	High	3 months	MSM	Sexual health clinic	No	Self-initiated	No
12	M	68	Recruited via charity	4 years and 8 months	MSM	Hospital (inpatient)	Attended A&E and GP prior to testing. On re-contacting GP he was directly admitted to hospital and tested.	HCP	Yes

As seen in Table 2, five SUs, all identifying as MSM, were tested for HIV at their first point of HCP contact in sexual health clinics and diagnosed at an early stage of disease. The remaining SUs were tested in hospital settings or in primary care (private general practitioner (GP)), largely following multiple previous attendance to primary care and A&E. Two SUs were seriously unwell at time of diagnosis and diagnosed with HIV in intensive care units (Table 2). Of the SUs tested in non-HIV specialist settings (primary care and hospital settings) all but one received a late HIV diagnosis.

### Theoretical framework

**Figure 1.**
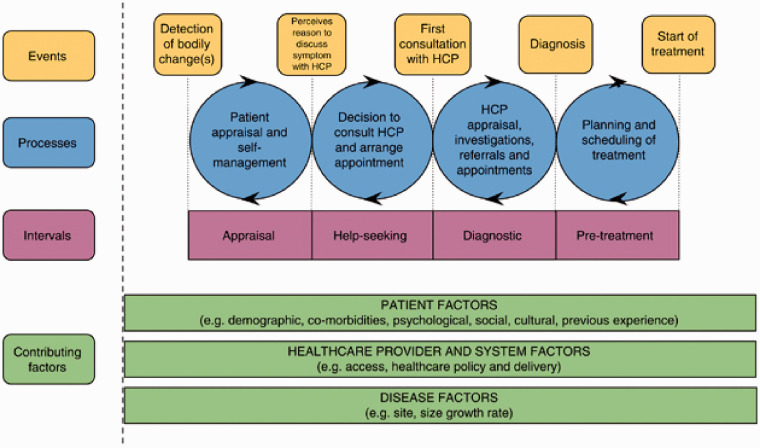
Model of pathways to treatment (MPT). Extracted from Walter et al. [15].

The Model of Pathways to Treatment (MPT) [15] was adopted as a theoretical framework to underpin this study (Figure 1). Pathway models, such as the MPT, aim to predict or explain health care-seeking behaviours through the exploration of health care-seeking as a journey, with defined processes and events leading to health service access and treatment [16].

The MPT was considered applicable in the context of HIV due to recognition within the model that symptoms of disease do not necessarily trigger health service access and that health problems may be asymptomatic. Someone with HIV can remain asymptomatic for up to 10 years from initial infection, therefore, other factors - such as awareness of engaging in ‘risky’ sexual behaviour - may prompt health care-seeking.

This study focuses on the following events outlined in the MPT: (i) detection of bodily changes, (ii) perceiving a reason to access health services, (iii) attendance at health services, and (iv) diagnosis [15]. Intervals exist between these events, during which multiple processes (cognitive, emotional, behavioural, organizational, or structural) may occur to hinder or promote the health care-seeking pathway [15]. Contributing factors, such as those related to the disease (e.g. symptom severity), the patient (e.g. previous experience of illness), and the provider (e.g. health care policy, access) influence the pathway.

### Interview design and conduct

Topic guides were used to assist the interviews. These were developed by all members of the research team following a review of the literature and were based around events in the MPT [15].

SU interviews focused on: (i) knowledge and awareness of HIV/AIDS prior to diagnosis, (ii) health service encounters preceding and at the point of testing positive for HIV, and (iii) perceptions of HIV prevention and testing services. HCP interviews focused on: (i) perceptions around the accessibility and acceptability of testing services to older adults, (ii) attitudes towards discussing HIV risk with older adults and offering a HIV test, and (iii) experiences of testing and diagnosing older adults with HIV.

Participants were offered the choice of being interviewed either within their own homes, at the University of York or in clinic, where this was possible. If a SU was interviewed in clinic this was timed around a pre-existing appointment for SUs and around workload commitments for HCPs. All interviews taking place on NHS sites were performed in private rooms, in comfortably furnished and quiet environments away from the clinical area. All interviews were audio-recorded and reflective notes were taken during interviews.

### Data analysis

Interviews were transcribed verbatim and were analysed thematically by SB using the stages of data familiarization, coding, and theme identification and refinement. The datasets were initially analysed separately to identify any distinctions between SU and HCP perceptions. Interviews were coded using initial codes generated from the interview topic guide and the events outlined in the MPT. Following the coding of each interview, codes were collated to generate broader themes. At this stage, mind maps were created for SU and HCP interviews to assist theme refinement. A matrix was also created, using events and processes within the MPT (to populate the columns of the matrix) and participants (to populate the rows) to help structure the analysis around the MPT. To enhance the rigour of the analysis, a sample of interviews were also analysed separately by JA and the coding approaches of SB and JA were discussed.

### Ethical approval

Ethical approval was granted by the Health Sciences Research Governance Committee at the University of York, the North East-York Research Ethics Committee (15/NE/0040), and Research and Development departments at each NHS recruitment site.

## Findings

SUs diagnosed at a late stage of disease often experienced non-linear journeys to diagnosis, involving missed opportunities for HIV testing and late presentation at health services. We identified contributing factors that affected events within the MPT (detection of bodily changes, perceiving a reason to access health services, attendance at health services and diagnosis) and therefore clinical stage of HIV infection at time of diagnosis.

Pathways to diagnosis were affected by 6 factors: (i) the non-specific nature of HIV symptoms and their misattribution as being age-related; (ii) symptom severity, impact, and visibility; (iii) HIV health literacy; (iv) perceptions of HIV risk; (v) geographical location; and (vi) assessment in non-specialist settings.

### Detection of bodily changes

#### Non-specific nature of HIV symptoms and their misattribution as being age-related

When symptomatic at their first contact with a clinician, SUs often presented with symptoms that were varied and non-specific in nature, including cough, headache and stomach problems. On experiencing symptoms, SUs drew upon their knowledge on health and illness to explain these changes. This included knowledge of symptomatology as well as knowledge of the risk factors for conditions. In most cases, symptoms were initially explained in terms of commonplace conditions or normalized as due to ageing:

I suppose people sort of think, ‘I just, you know, can’t shake this cold, that’s cos, you know, I’m getting older.’ All these sorts of things when actually these are things that potentially could be associated with HIV (HCP11)

Although most SUs had symptoms at the point of accessing health services, four MSM were diagnosed at an early stage of disease and were asymptomatic at presentation (Table 2). MSM that were asymptomatic at diagnosis had either sought a HIV test due to concerns around potential HIV exposure or as part of a routine sexual health screen. The participants identifying as MSM in the study tended to have a good knowledge around HIV prior to diagnosis, but this awareness did not necessarily lead to greater HIV risk avoidance. This was a finding corroborated by HCPs in their experience of caring for older MSM with HIV.

### Perceiving a reason to access health services

Presence of symptoms or engagement in HIV risk behaviours (e.g. condomless sex) may not be enough to encourage attendance for testing. In considering symptomatic and asymptomatic SUs, the following also affected the decision to access health services.

#### Symptom severity, impact, and visibility

For symptomatic SUs, health care-seeking was prompted when symptoms affected their ability to perform day-to-day activities, causing significant disruption to their lives:

[I had] a cough on my chest, [a] constant cough and then I’m out of breath. If I’m talking, I’m out of breath. If I were talking like this, I’d be out of breath and coughing again (SU4)

When symptoms were experienced with greater severity, and occurred over a longer period than previously endured, this also prompted health care-seeking. The recognition of symptoms by others, such as family and friends, also served to prompt health service access. As symptoms persisted, the need to identify an underlying cause and receive symptom relief became increasingly urgent. This resulted in SUs attending HCPs multiple times, looking for answers:

Eventually I went back [to the GP] and said, ‘I can’t deal with it anymore.’ (SU2 - attended a GP four times before being diagnosed in A&E)

#### HIV health literacy

In the absence of symptoms, health care-seeking decisions were based on ‘external cues’ to action such as knowledge about HIV. Generally, adults aged 50+ years were considered by HCPs to have lower levels of HIV knowledge than younger people. HIV and sexually transmitted infection (STI) prevention messages were often felt by HCPs and SUs to exclude adults aged 50+ years, through a youth focus and use of communication mediums associated with younger people. This was reported by HCPs to leave a gap in knowledge surrounding the importance of practising safer sex in older age:

I mean, there is that, the sort of evidence around men coming out of long term relationships, being married for thirty years and then divorcing and getting into new relationships and then just not necessarily reconnecting with the kind of, the safe sex message they had when they were like an adolescent or in their early twenties (HCP7)

It was considered by HCPs that since the 1980s, heterosexuals, particularly those not targeted by HIV campaigns (i.e. not of black African ethnicity or identifying as MSM), had received very little information about HIV/AIDS. In their experience, HCPs reported that HIV knowledge among older heterosexual men and women diagnosed with HIV was largely based on the 1987 ‘*AIDS: Don’t Die of Ignorance*’ campaign and storylines in British TV soap operas and American films, such as *EastEnders* (first episode broadcast in 1985) and *Philadelphia* (released in 1993), involving HIV positive characters. These reference points of knowledge are from a time prior to the development of effective treatment, and up-to-date knowledge of treatment advancements may therefore be lacking.

MSM SUs in the study reported and demonstrated high levels of HIV knowledge, a finding corroborated by HCPs. HCPs considered that in their experience MSM tended to receive more up-to-date and factual HIV information and be better informed than heterosexual men and women. HIV knowledge in MSM participating in the study came from being openly gay and actively engaged with the gay community, previous voluntary work with HIV charities, and personal experience in knowing someone with HIV:

[In the] gay community here [a high HIV prevalence area] … you go to a bar, there’s information leaflets. I mean, you speak with friends, all ages, and diversities … all of the information is there, you know, to reach to all, a diverse group of gay people (SU7)

Extensive knowledge of HIV, including common symptomatology and perceived risk status, enabled one MSM SU to recognize his bodily changes as ‘classic symptoms’ of HIV infection. This person's awareness of HIV was based on caring for a partner who died early in the UK epidemic, prior to the development of effective treatment.

#### Perceptions of risk

In conjunction with HIV knowledge, HCPs considered that the perceptions of personal susceptibility to HIV were lower in groups not targeted by HIV prevention campaigns. This was also expressed in interviews with SUs. The continual representation of MSM in HIV campaigns served as an ongoing cue to health care-seeking for this group and was linked by HCPs to lower risk perception in heterosexual males:

There is still that culture that straight people think [HIV] only happens to the gay people (HCP3)

In interviewing a white heterosexual female SU, it was clear that she had never considered herself, or anyone she considered demographically like her, to be at risk of HIV. The shock this SU felt at diagnosis appeared much greater than among the interviewed heterosexual male and MSM SUs:

Like I say, you think junkie, you know, gay and prostitutes. It was the worst three things you could be named (SU2)

HCPs acknowledged that SUs receiving a late HIV diagnosis often did not belong to groups that would be broadly considered at risk of HIV, either by the individual receiving the diagnosis or by clinicians:

Often in our experience it’s the … unusual people that don’t get offered a HIV test, because nobody kind of thinks that could be what it is, so they tend to focus on the people traditionally that they would think has HIV (HCP4)

HCP also discussed that older age may produce a false sense of security against STI/HIV acquisition.

Although belonging to a group targeted by HIV testing and prevention campaigns, some MSM SUs, notably those that did not attend sexual health services as the first point of HCP contact, discussed an altering in their perceptions of personal HIV risk with advancing age. Diminished personal risk perception with time appeared as what the researchers considered to be a sort of ‘precaution fatigue’; users described growing tired of taking such stringent measures to prevent HIV and the years spent living in fear of HIV/AIDS:

People like me that have grown up with HIV, you know, tended to be very, very careful about it, you know, for many, many years that in age and I think there’s probably a lot of people like me that in age, with age, you know, your level of carefulness drops (SU11)

### Attendance at health services and diagnosis

#### Assessment at non-specialist settings

For SUs that attended a GP as their first health care contact, there were often multiple stages before HIV diagnosis, with back and forth general practice and secondary care attendances (Table 2). In some situations, SUs that were diagnosed late were misdiagnosed and treated for different conditions in non-HIV specialist settings before being tested for HIV, despite presenting with conditions suggestive of HIV infection in high risk groups (for example, pneumonia in MSM) [8]. In most instances, however, SUs presented with symptoms that could be associated with a range of other more common health problems. In that case, unless attending a sexual health/genito-urinary medicine (GUM) clinic, HCPs voiced that HIV was unlikely to be considered as a probable diagnosis until a range of other avenues had been exhausted:

A lot of the symptoms for HIV … are …  not so obvious that they’re kind of distinctive* … *.Understandably perhaps, doctors can miss these things, but I guess … they do tend to actually be more likely to test certain people and not others - so they must have certain prejudices to some extent in their own kind of thinking about HIV and who has it and who doesn’t (HCP4)

SUs expressed greater confidence in the ability of HCPs working in sexual health/GUM settings to provide HIV testing than GPs, or other non-HIV specialists. A minority of HCPs expressed the belief that GPs would prefer for people to access a sexual health/GUM services, as it was not their responsibility to test.

It was considered by HCPs that GPs may lack the competency and confidence to provide testing. HCPs perceived that in non-specialist settings, clinicians feel less comfortable about discussing sexual health and HIV with their older patients. It was also perceived that clinicians in non-specialist settings were considered by HCPs as more likely to attribute symptoms to more common conditions.

Unless attending a sexual health/GUM clinic, HCPs considered that HIV was unlikely to be considered as a probable diagnosis until a range of other avenues had been exhausted.

Advancing age was reported by HCP to reduce the likelihood of being considered at risk of HIV by non-HIV specialists, with the misconception that older adults do not engage in high risk behaviours for HIV. For adults not belonging to targeted groups, HCPs considered that advancing age compounded their likelihood of not being considered at risk. HCPs considered older heterosexual females, particularly of white ethnicity, as least likely to be offered testing. MSM were considered most likely to be offered testing.

Several HCPs reported that sexual health/HIV services are youth-focused, which could serve as a barrier to older people attending. This was expressed in terms of the environment, rather than the care provided in these settings. It was reported by HCPs as routine practice for younger people to attend sexual health/GUM services and be screened for STIs. However, HCPs and SUs did not consider this the case for older adults, aside from MSM:

If you are not active on the gay scene … the difficulty is, it’s getting through that door. Not everybody checks regularly (SU6)

#### Geographical location

All SUs that reported previous sexual health/GUM clinic attendance, or self-initiated testing in these settings, lived in high HIV prevalence areas. Several SUs felt that it would be easier to access a sexual health clinic in an urban, high HIV prevalence area than a rural, low prevalence area. This was discussed in terms of travel time.

It was also considered that the limited choices of where to seek sexual health care in rural or suburban low prevalence areas might create greater concerns regarding confidentiality. It was clear that this concern could lead to some people travelling further away from their home for testing. This carries cost and time implications and could serve as a testing barrier. It was also considered that it would be less embarrassing for older people to attend sexual health/GUM clinics in high, rather than low, HIV prevalence areas. This was due to the wider age range of attendees in these settings, a result of higher levels of STIs and HIV:

The majority are young people, who I would guess attend sort of the more suburban sexual health clinic. You know, probably here in [a high prevalence area] you’d have gay men of all ages (HCP1)

MSM that attended sexual health/GUM services to self-initiate testing perceived this environment to be readily accessible. This was linked to the friendliness of HCPs working in this setting:

In my book already, you know this is a fantastic institution. They make it very easy to come and test, yeah. So they really accommodate you and they’re friendly and they’re nice and they take their time to chat and explain and you know, develop a relation to them to some extent (SU11)

## Discussion

This paper has identified several barriers to the early detection of HIV in adults aged 50+ years, including: absence/non-specificity of symptoms, low levels of health literacy on HIV, low perception of risk in both service users and health care professionals, and sexual health services oriented towards younger people. These barriers help to explain the relatively low levels of uptake of HIV testing we have previously described in older age groups [11], despite rising infection rates.

Given that symptoms are often an internal cue or trigger for health care-seeking [17], a major barrier to early HIV diagnosis was the absence of symptoms, or a lack of concerning symptoms, potentially until several years after infection. Indicator conditions to prompt HIV testing (for example, pneumonia) did not do so, often due to their non-specificity. This is applicable to the early diagnosis of HIV at all age ranges. Previous research has indicated that GPs are driven by ‘patterns’ in symptoms [18,19] and diagnostic errors can occur when symptoms do not follow a pattern recognized by a GP [18]. GPs’ prioritization of alternative diagnoses is likely to reflect the relatively uncommon occurrence of HIV in the general population over age 50. This barrier is a particular problem in these older age groups because symptoms such as tiredness and weight loss may be attributed to ‘normal' ageing [20]. This may lead to delays in accessing health services and in HCPs offering a test. On the other hand, the specialist providers interviewed in this study have greater experience and familiarity with the diagnosis of HIV.

SU age was also reported as a barrier to HCPs discussing sexual health/HIV with older people. This is in concordance with previous research that reports GP embarrassment and worries about causing offence in discussing sexual health with adults aged 50+ years [21]. Further, HCPs are less likely to take a sexual history into account and discuss sexual health/HIV with older people [22,23].

Perceived susceptibility to an illness is widely regarded as a driver to health care-seeking [24]; however, most SUs had not considered HIV as a possible diagnosis. Personal perceptions of HIV risk and sexual health/HIV knowledge have been reported as lower in the over 50 s than younger adults [25]. Sexual health/HIV policies and campaigns in the UK have largely focused on men who have sex with men [26] and black African men and women [27], and these campaigns have tended to focus on younger people [28]. In contrast to younger adults, there has only been one sexual health campaign, the *Middle-age Spread* campaign, aimed at older adults in the UK [29]. This has arguably created the impression that sexually transmitted infections and HIV are not of concern for the over 50 s, or those not belonging to ‘high risk’ groups. An unintended consequence of this approach appears to be that those not targeted are at increased risk of late diagnosis.

Higher levels of HIV knowledge were found in MSM, associated with the targeting of this group in HIV prevention and testing messages [26,30]. MSM were generally considered more likely to attend sexual health/GUM services than heterosexuals, and to request routine sexual health screening. For several MSM, their perception of personal HIV risk and measures to protect themselves against HIV had diminished with advancing age, an occurrence which we have termed ‘precaution fatigue’. A lessening of precaution efforts and diminished perception of personal risk after years of living in fear of HIV, and not becoming infected, has created a false sense of safety.

### Limitations

The ability to recruit 12 SUs for in-depth interviews, given difficulties in accessing this group due to the stigma and discrimination still experienced by people living with HIV, was a major strength of this study. The quality of the study was enhanced through he use of interviews with both SUs and HCPs to corroborate findings. However, there were some limitations in our recruitment. Overall, patterns in SU recruitment largely reflected wider trends in HIV epidemiology; new HIV diagnoses in older adults are largely in people in their fifties, and MSM are most affected by HIV. The highest number of people living with HIV are in London. Despite attempts to recruit participants from several sites, most SUs were from one NHS site in a high HIV prevalence area and this limits the transferability of the findings beyond high prevalence areas, particularly outside of London.

SUs were also not diverse in terms of ethnicity or sexual orientation, and most people were aged 50–53 years – that is, at the youngest range of eligibility to participate. Therefore, little insight was gained into the experience of receiving a diagnosis beyond this age, or of a heterosexual man or woman, or someone from a non-White ethnic background being diagnosed with HIV. By recruiting SUs that were initially approached by sexual health/HIV clinicians, this may have also introduced a bias in who took part in the research. We may have been more likely to have spoken with SUs that had a good relationship with their clinicians and felt obliged to discuss their experiences within HIV services in a positive light.

Interviews with HCPs was limited to specialist services and further qualitative research with GPs and non-HIV specialists would be valuable to gain their perspectives on barriers to testing older adults for HIV. Qualitative research with GPs is particularly important given that they are often the first point of health care contact and are key to promoting earlier HIV detection in older adults.

## Conclusion

Older adults face additional barriers to HIV testing than younger people, especially when they do not belong to a group targeted by HIV prevention and testing campaigns. In order to diagnose HIV more promptly in adults aged 50+ years, HIV knowledge and risk perception must increase in both older people and health care professionals. For most service users their first health care contact was with a GP, highlighting their pivotal position to promote early HIV detection. GPs and other non-HIV specialists are critical in the challenge to provide equitable HIV testing beyond the traditional ‘high risk’ groups.
